# Diversification of Gene Expression during Formation of Static Submerged Biofilms by *Escherichia coli*

**DOI:** 10.3389/fmicb.2016.01568

**Published:** 2016-10-05

**Authors:** Olga Besharova, Verena M. Suchanek, Raimo Hartmann, Knut Drescher, Victor Sourjik

**Affiliations:** ^1^Max Planck Institute for Terrestrial MicrobiologyMarburg, Germany; ^2^LOEWE Center for Synthetic Microbiology (SYNMIKRO)Marburg, Germany; ^3^Zentrum für Molekulare Biologie der Universität Heidelberg, DKFZ-ZMBH AllianceHeidelberg, Germany

**Keywords:** bacteria, matrix, gene expression, curli, motility, flagella, stationary phase, growth

## Abstract

Many bacteria primarily exist in nature as structured multicellular communities, so called biofilms. Biofilm formation is a highly regulated process that includes the transition from the motile planktonic to sessile biofilm lifestyle. Cellular differentiation within a biofilm is a commonly accepted concept but it remains largely unclear when, where and how exactly such differentiation arises. Here we used fluorescent transcriptional reporters to quantitatively analyze spatio-temporal expression patterns of several groups of genes during the formation of submerged *Escherichia coli* biofilms in an open static system. We first confirm that formation of such submerged biofilms as well as pellicles at the liquid-air interface requires the major matrix component, curli, and flagella-mediated motility. We further demonstrate that in this system, diversification of gene expression leads to emergence of at least three distinct subpopulations of *E. coli*, which differ in their levels of curli and flagella expression, and in the activity of the stationary phase sigma factor σ^S^. Our study reveals mutually exclusive expression of curli fibers and flagella at the single cell level, with high curli levels being confined to dense cell aggregates/microcolonies and flagella expression showing an opposite expression pattern. Interestingly, despite the known σ^S^-dependence of curli induction, there was only a partial correlation between the σ^S^ activity and curli expression, with subpopulations of cells having high σ^S^ activity but low curli expression and *vice versa*. Finally, consistent with different physiology of the observed subpopulations, we show striking differences between the growth rates of cells within and outside of aggregates.

## Introduction

The majority of bacteria can grow on different surfaces into biofilms, which are multicellular communities that are embedded in self-produced extracellular matrix (Hall-Stoodley et al., 2004; O’Toole et al., 2000; López et al., 2010; Häussler and Fuqua, 2013). Several different experimental models have been used to simulate diverse natural biofilms (Serra and Hengge, 2014). Biofilms grown in microtiter dishes or flow chambers represent structures that are formed in aquatic environments (Pratt and Kolter, 1998; Danese et al., 2000; O’Toole, 2011). Under static conditions in liquid media, i.e., without flow, bacteria frequently form not only the submerged biofilms that grow at the bottom of the well, but also pellicles at the liquid-air interface (Hung et al., 2013; Vlamakis et al., 2013). Alternatively, submerged biofilms can develop under flow conditions, where nutrients are constantly resupplied (Sternberg et al., 1999; Teal et al., 2006). Finally, macrocolony biofilms formed on agar plates represent another biofilm model that mimics natural communities growing on organic material (Serra et al., 2013b).

The formation of three-dimensional (3D) structures in biofilms is a highly regulated and dynamic process that is influenced by many factors (Flemming and Wingender, 2010). It critically depends on production of the extracellular matrix, which can vary greatly between biofilms of different species and environmental factors, but typically includes polysaccharides, protein filaments, lipids and nucleic acids (Branda et al., 2005; Flemming and Wingender, 2010; McCrate et al., 2013; Hobley et al., 2015; Hufnagel et al., 2015). Consequently, regulation of genes involved in matrix production represents a critical step in the lifestyle transition from the motile planktonic state to the sessile biofilm state. Similarly important during this transition might be the regulation of motility genes, as motility is typically required for the initial surface attachment and biofilm formation but is turned off in mature biofilms (Pratt and Kolter, 1998; Domka et al., 2007; Vlamakis et al., 2008; Hung et al., 2013).

*Escherichia coli* was one of the first established model organisms for studies of biofilm formation. A number of factors including proteinaceous curli fibers (Römling et al., 1998b; Vidal et al., 1998; Prigent-Combaret et al., 2000; Römling, 2005), type I fimbriae (Schembri and Klemm, 2001), Antigen 43 (Ag43) (Danese et al., 2000; van der Woude and Henderson, 2008), poly-β-1,6-*N*-acetyl-D-glucosamine (PGA) (Wang et al., 2004), and colanic acid (Beloin et al., 2008) have been implicated in surface-attachment, cell-cell interactions and microcolony formation of *E. coli* (Beloin et al., 2008). Flagella and motility were also shown to influence biofilm formation, either by enhancing attachment (Pratt and Kolter, 1998; Friedlander et al., 2015), mediating surface sensing that triggers matrix production (Belas, 2013, 2014), or being involved in the biofilm architecture (Wood et al., 2006; Serra et al., 2013b). Finally, cellulose, which is commonly produced in wild isolates of *E. coli*, can also contribute to the overall biofilm architecture (Zogaj et al., 2001; Serra et al., 2013b). As in other bacteria, matrix composition – and therefore structural organization – of *E. coli* biofilms are affected by environmental conditions such as temperature, availability of nutrients, and shear forces due to flow (Römling et al., 1998b; Sutherland, 2001; Serra and Hengge, 2014; Persat et al., 2015).

Global changes in gene expression that take place during the development of *E. coli* biofilms have so far mainly been studied on whole communities (Schembri et al., 2003; Beloin et al., 2004), which is likely to obscure the heterogeneity in cellular states that is inherent to most biofilms (Stewart and Franklin, 2008; Serra et al., 2013b; van Gestel et al., 2015). It is generally assumed that the transition of *E. coli* from the planktonic state towards the biofilm lifestyle must include the regulation of flagellar genes as well as genes that are expressed at entry into the stationary phase under control of the general stress response sigma factor σ^S^ (Hengge-Aronis, 2002; Hengge and Storz, 2011). Among other genes, σ^S^ positively regulates CsgD, the master transcriptional regulator that promotes production of curli, a major proteinaceous matrix component of *E. coli* biofilms grown at low temperature (≤30°C) (Olsén et al., 1989; Hammar et al., 1995; Römling et al., 1998b; Brown et al., 2001; Ogasawara et al., 2010b). CsgD also positively controls cellulose synthesis (Römling et al., 2000; Brombacher et al., 2003). Consistent with its function during the lifestyle switch, the expression of matrix components and flagella is inversely regulated in *E. coli* through several mutual inhibitory connections between σ^S^ and the flagellar regulon (Pesavento et al., 2008; Povolotsky and Hengge, 2012). Such inverse regulation is consistent with the global pattern of flagella and curli production within macrocolony biofilms (Serra et al., 2013b). However, global control via σ^S^ cannot fully explain the transition towards biofilm formation, as stationary phase planktonic cells differ significantly from cells associated in multicellular biofilm communities (Schembri et al., 2003; Mikkelsen et al., 2007; White et al., 2010).

In this study we quantitatively analyzed spatio-temporal changes in expression of several key groups of *E. coli* genes during formation of submerged biofilms and pellicles in an open static system. The expression of genomic fluorescent reporters was studied at a single-cell level using both flow cytometry and image analysis of the biofilm structures. We show that while expression of the curli and flagellar genes is confined to different subpopulations of cells, other gene classes are expressed more uniformly across the subpopulations. Furthermore, using molecular timers we demonstrate that growth rates of cells vary between different regions of the biofilm.

## Materials and Methods

### Bacterial Strains, Plasmids, and Media

The strains and plasmids used in this study are listed in Supplementary Table 1. All strains were derived from *E. coli* W3110 (Serra et al., 2013b). Cells were grown in tryptone broth (TB) medium (10 g tryptone, 5 g NaCl per litre) supplemented with antibiotics, where necessary. Gene deletions were obtained via PCR-based inactivation of chromosomal genes (Datsenko and Wanner, 2000) or using P1 transduction (Miller, 1972). Km^r^ cassettes were eliminated via FLP recombination (Cherepanov and Wackernagel, 1995). For the construction of pOB2 and pVM42, *mCherry* and *egfp* genes were amplified by PCR using a forward primer containing the artificial strong ribosome binding site (RBS) ACAACTTAAGGAGGTATTC (Salis et al., 2009) and cloned into the pTrc99a vector with *Kpn*I/*Hin*dIII.

### Construction of Transcriptional Genomic Reporters

For the construction of transcriptional genomic reporters derivatives of plasmid pKD13 (Datsenko and Wanner, 2000) were generated. For this purpose either Gibson assembly or circular polymerase extension cloning (Quan and Tian, 2009) were applied. All fragments including linearized vector with kanamycin cassette flanked by FLP recombination target sites, RBS, fluorescent protein and gene sites for insertion were first amplified using Phusion Polymerase and then assembled into one circular DNA fragment in a single reaction. In this way, constructs with an additional RBS (for transcriptional reporters), *mCherry* or superfolder GFP (*sfGFP*) genes and Km^r^ cassette flanked by the homologous regions for genomic insertion were created. The lambda Red recombination system (Datsenko and Wanner, 2000) was used to introduce fluorescent reporters into native chromosomal loci via homologous recombination. Positive clones were checked by PCR for the correct insertion using specific primers and subsequently sequenced. After Km^r^ cassettes were eliminated via FLP recombination (Cherepanov and Wackernagel, 1995), a second reporter was introduced in its native chromosomal locus.

### Biofilm Growth and Analysis

For the analysis of *E. coli* biofilm formation, overnight cultures grown in TB in a rotary shaker at 30°C were diluted 1:100 into fresh TB medium and grown at 200 rpm to the mid-exponential phase (OD_600_ = 0.6) at 30°C. The samples were further diluted to an OD_600_ of 0.05 in TB medium and 400 μl of this suspension was seeded per well into the 8-well microscope slides with untreated surfaces (μ-Slide, 8-well untreated bottom, ibidi GmbH, Germany). TB was supplemented with 100 μg/ml ampicillin (Amp) and 10 μM isopropyl-β-D-thiogalactopyranosid (IPTG) when cells carried either plasmid pVM42 encoding *egfp* or pOB2 encoding *mCherry* for visualization of all cells in the biofilm. Biofilms were grown at 30°C without shaking for the indicated time. Gene expression and/or structure of undisrupted biofilms were visualized using confocal microscopy. In parallel, biofilms were separated into pellicle, supernatant and surface-attached cell fractions and subjected to flow cytometric single cell analysis.

In addition, biofilm formation on microtiter plates was quantified using crystal violet (CV) assays (O’Toole, 2011). For this purpose, overnight cultures were diluted 1:100 in fresh TB medium, regrown until OD_600_ of 0.6, and adjusted to OD_600_ 0.05. Three hundred microliter of this suspension was then loaded into a 96-well plate (Corning Costar, flat bottom, Sigma Aldrich, Germany). After 24 h of incubation at 30°C, the OD_600_ of samples was measured, non-attached cells were removed from wells and wells were intensively washed with water. Cells bound to the polysterene plastic walls were subsequently stained with 1% CV solution, 300 μl per well for 20 min at room temperature. Then, CV was removed, wells were washed three times with water and then the plate was left to dry for 1 h. Remaining CV stain in attached cells was solubilized by adding 300 μl of 96% ethanol and measured at OD_595_. Finally, all CV values were normalized by the respective OD_600_.

### Flow Cytometry

Cells were collected from different biofilm fractions at the indicated time points of biofilm growth, washed two times and resuspended in phosphate-buffered saline (PBS) (8 g NaCl, 0.2 g KCl, 1.44 g Na_2_HPO_4_, 0.24 g KH_2_PO_4_) prior to flow cytometric analysis using BD LSRFortessa SORP cell analyzer (BD Biosciences, Germany). The pellicle fraction was collected by taking the thin film layer from the liquid/air interface using a pipette and resuspended in 1 ml PBS. After pellicle removal, 100 μl of culture medium was collected as the supernatant fraction. The rest of the supernatant was discarded and the remaining surface-attached cells were washed two times with PBS. Subsequently, surface-attached cells were scraped from the well surface using a 1 ml pipette tip and vigorously vortexed before the measurements to disrupt cell aggregates. The completeness of aggregate disruption was confirmed by flow cytometry, using fortward scatter (FSC) and side scatter (SSC) parameters. Data were analyzed using FlowJo software version 10.1r5 (FlowJo LLC, Ashland, OR, USA), applying a software-defined background fluorescence subtraction.

### Confocal Microscopy

*Escherichia coli* intact biofilms were visualized using an inverted Zeiss Axio Observer Laser Scanning Microscope (LSM) 880 equipped with a C-APOCHROMAT 40x/1.2 Water Corr-UV-VIS-IR objective. The following lasers were used: 458 nm, 488 nm, 514 nm Argon laser and 561 nm DPSS laser. Each experiment was performed at least three times on independent biological replicates. Obtained images and *z*-stack projections were visualized using Zeiss ZEN System imaging software (Zeiss). Thickness of pellicles was measured using an upright Leica TCS SP5 confocal laser scanning microscope equipped with HC PL FLUOTAR 10x/0.3 dry objective.

### Quantitative Image Analysis

Quantitative image analysis was performed using Matlab (Mathworks) in several steps. First, image stacks showing constitutive fluorescence signal were up-sampled along the *z*-axis to obtain equal voxel side lengths. Second, noise was reduced by convolution with a small averaging filter kernel. Third, segmentation was performed by 3-level thresholding according to Otsu’s Method (Otsu, 1979) where class one was assigned to background, while class two and three where treated as foreground. Finally, the obtained binary image stack was dissected into cubes (side length: 5 px, approximately 1 μm). In cases where no constitutive fluorescence marker was available, the image stacks of each fluorescence channel were segmented individually and the volumetric data was merged before dissection (see Supplementary Figure 8). For each dissected volume the underlying unprocessed fluorescence intensities of GFP and mCherry were measured. In addition, the local cell density was determined by measuring the occupied volume inside a shell of 3 μm radius around the center of mass of each cube. Paraview was used for 3D-visualization (Ahrens et al., 2005; Flandin, 2015).

## Results

### Curli and Flagella are Required for Formation of *E. coli* Submerged Biofilms

**FIGURE 1:**
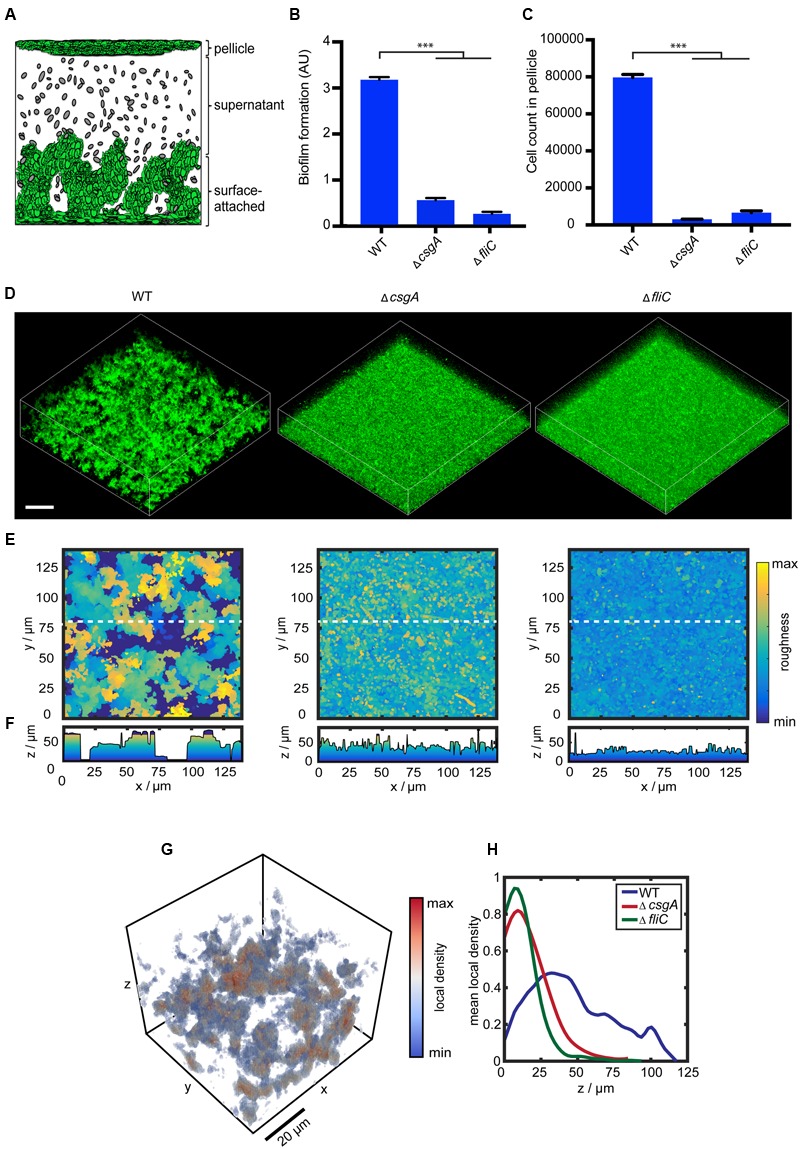
**Curli fibers and flagella are required for structure formation in submerged *Escherichia coli* biofilms. (A)** Schematic representation of submerged *E. coli* biofilms grown in microtiter plates, indicating three fractions corresponding to the pellicle, supernatant and surface-attached cells. Note that the structures are not drawn to scale (see text for details). **(B)** Biofilm formation by wild-type strain W3110 and mutants lacking either curli (Δ*csgA*) or flagella (Δ*fliC*) that were grown under static conditions at 30°C for 24 h. Biofilm formation was quantified using crystal violet (CV) staining, with CV values normalized to the optical density shown in arbitrary units (AU). ^∗∗∗^indicates *P* < 0.0001 according to unpaired *t*-test. Standard errors from three independent experiments are indicated. **(C)** Number of cells counted per 60 s in the pellicle fraction of submerged biofilms, measured using flow cytometry. **(D)** Confocal fluorescence microscopy images (maximum projection) of eGFP-expressing cells (carrying pVM42) in static biofilms grown in microtiter plates at 30°C for 24 h. Scale bar, 40 μm. **(E)** Top views and corresponding **(F)** roughness profiles of submerged biofilms shown in **(D)**. The color scale shows roughness values from lowest (blue) to highest (yellow). **(G)** Visual representation of calculated local density (see Materials and Methods for details) for a subset of the intact submerged biofilm shown in **(D)**. As indicated by the color scale bar, red values correspond to dense regions, while blue values represent regions with low local density. **(H)** Profiles of mean local density along the biofilm height (*z*-axis) for W3110 (WT; blue), Δ*fliC* (green), and Δ*csgA* (red).

As a model to investigate changes in gene expression during formation of *E. coli* biofilms, we used the biofilm assay in microtiter plates under static conditions (i.e., without flow) at 30°C. Already after 6 h, and even more evident after 24 h, submerged biofilm structures of 50-60 μm thickness could be observed in this assay on the bottom of the well, and a pellicle of 10-15 μm thickness was formed at the liquid-air interface (**Figure 1A**). Taking into account the heterogeneity of such a static biofilm system, for our subsequent gene expression analysis using flow cytometry, we separated three physiologically different fractions: cells within the pellicle, cells attached to the surface of the well, and the individual cells and small aggregates that remain outside of these structures (termed “supernatant” in this article) (see Materials and Methods for details).

Confirming previous observations (Pratt and Kolter, 1998; Vidal et al., 1998), we observed that formation of biofilms under these conditions requires curli fibers and flagella, since deletion of major subunits of either curli (Δ*csgA*) or flagella (Δ*fliC*) filaments led to large decrease of the biomass of submerged biofilms as quantified by CV staining (**Figure 1B**). Similarly, pellicle formation was completely abolished, as quantified by measuring cell density at the liquid-air interface using flow cytometry (**Figure 1C**).

Confocal laser scanning microscopy further revealed that both curli fibers and flagella are required for the 3D structure formation (**Figure 1D**). Quantitative analysis of the microscopy images (see Materials and Methods for details) confirmed that both Δ*csgA* and Δ*fliC* strains do not form mature 3D structures, but display a uniform surface roughness profile in contrast to the wild type (**Figures 1E,F**). In order to further distinguish cells associated with structures and aggregates from single cells, we calculated the local cell density within the microscopy images (**Figure 1G**). The local density profile for the wild-type strain differed significantly from both mutant strains (**Figure 1H**). Whereas the wild type shows regions of high local density up to 100 μm above the surface, which correspond to cell aggregates and/or microcolonies that are visible in confocal images (**Figure 1D**; Supplementary Figure 1A), both Δ*csgA* and Δ*fliC* strains show a high density only at the bottom of the well, which is likely to represent sedimented individual cells or cells being attached to the surface.

Notably, both curli and flagella also provide structure to the macrocolony biofilms grown on the soft agar (Serra et al., 2013a,b), suggesting similarity between these two very different types of multicellular communities formed by *E. coli*. Among other tested potential matrix components or adhesins (Korea et al., 2010), only a lack of fimbriae showed more than 50% reduction in biofilm formation under our conditions, as quantified by CV staining (Supplementary Figure 1B). Interestingly, this defect was only observed for CV staining of the biomass but not in microscopy images (Supplementary Figure 1C), indicating that fimbriae are required for surface attachment but not for structure formation.

### Curli and Flagella Show Mutually Exclusive Expression

**FIGURE 2:**
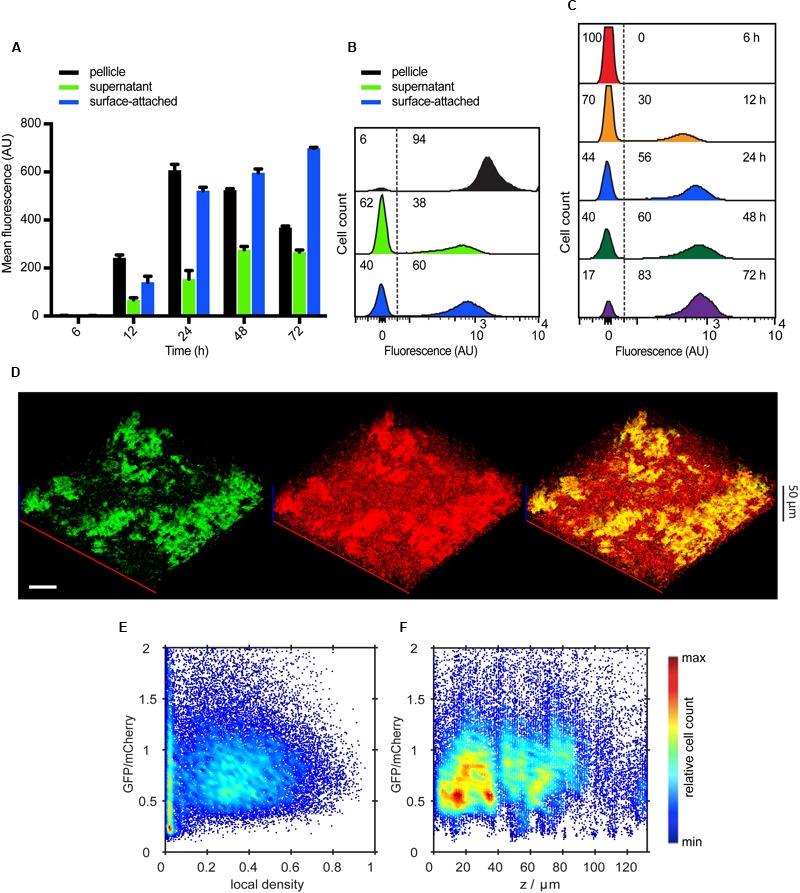
**Spatio-temporal expression of curli fibers in static submerged biofilms. (A)** Mean of P*csgA*-sfGFP fluorescence intensity measured in fractions corresponding to pellicle, supernatant and surface-attached cells (shown in black, green and blue, respectively, as indicated) at selected time points by flow cytometry. Shown are mean values calculated from three independent experiments and corresponding standard errors. **(B)** Flow cytometry analysis of single-cell distributions of P*csgA*-sfGFP fluorescence in individual biofilm fractions at 24 h. **(C)** Changes in the single-cell expression of curli measured over time in surface-attached cells. Numbers in **(B,C)** indicate the percentage of curli-OFF and curli-ON cells (separated by dashed line). Negative values in **(B,C)** arise due to background subtraction (see Materials and Methods); for data visualization, biexponential transformation was applied to scale the data on the *x*-axis. **(D)** Confocal fluorescence microscopy images (maximum projection) showing expression of P*csgA*-sfGFP (green) in biofilms at 24 h. For a reference, all cells were labeled with constitutively expressed mCherry (red) (pOB2). Scale bar, 40 μm. **(E,F)** Expression profile of P*csgA*-sfGFP in confocal images as shown in **(D)**, as a function of local density **(E)** and along the height (*z*-axis) of the biofilm **(F)**. Values of GFP fluorescence are normalized to fluorescence of mCherry which labels all cells. The color scale shows relative cell count from lowest (blue) to highest (red).

We next analyzed the expression pattern of curli and flagellar genes during transition towards the biofilm growth. For this purpose we constructed transcriptional reporters by integrating genes encoding superfolder GFP (sfGFP) or mCherry downstream of chromosomal *csgA* and *fliC*, respectively, and performed quantitative single cell analysis of gene expression in different biofilm fractions (**Figures 2A–C**) using flow cytometry. P*csgA*-sfGFP expression could be detected 12 h after planktonic cells were seeded onto microtiter plates and increased with time; its mean levels were significantly higher in the pellicle and the surface-attached cells (*P* < 0.0001 according to unpaired *t*-test), compared to the supernatant fraction (**Figures 2A,B**). Notably, P*csgA*-sfGFP expression was bimodal in all fractions (**Figure 2B**) and the increase in the mean fluorescence during biofilm development primarily occurred through an increase in the fraction of curli-ON cells (**Figure 2C**). Note that residual populations of curli-ON cells in the supernatant and of curli-OFF cells in the pellicle and surface-attached biofilm structures might be at least partly due to an imperfect separation of different fractions in our assay.

Confocal microscopy of intact 3D structures further confirmed bimodal expression of curli fibers in submerged biofilms, with high levels of P*csgA*-sfGFP being mostly confined in cell aggregates/microcolonies and little or no expression in other cells within the biofilm (**Figure 2D**). Consistently, quantitative image analysis (see **Figure 1G** and Material and Methods for details) showed high curli expression in regions of higher local density (**Figure 2E**). As already observed in flow cytometry data for the supernatant fraction (**Figure 2B**), the majority of cells in the regions of low local density showed little or no GFP expression and thus most probably represent individual curli-OFF cells. Notably, the expression profile of P*csgA*-sfGFP along the height of the submerged biofilm showed that the majority of cells expressing curli are found in a distinct region up to 40 μm from the bottom of the well (**Figure 2F**), which contains the majority of cell structures/aggregates (Supplementary Figure 1A).

**FIGURE 3:**
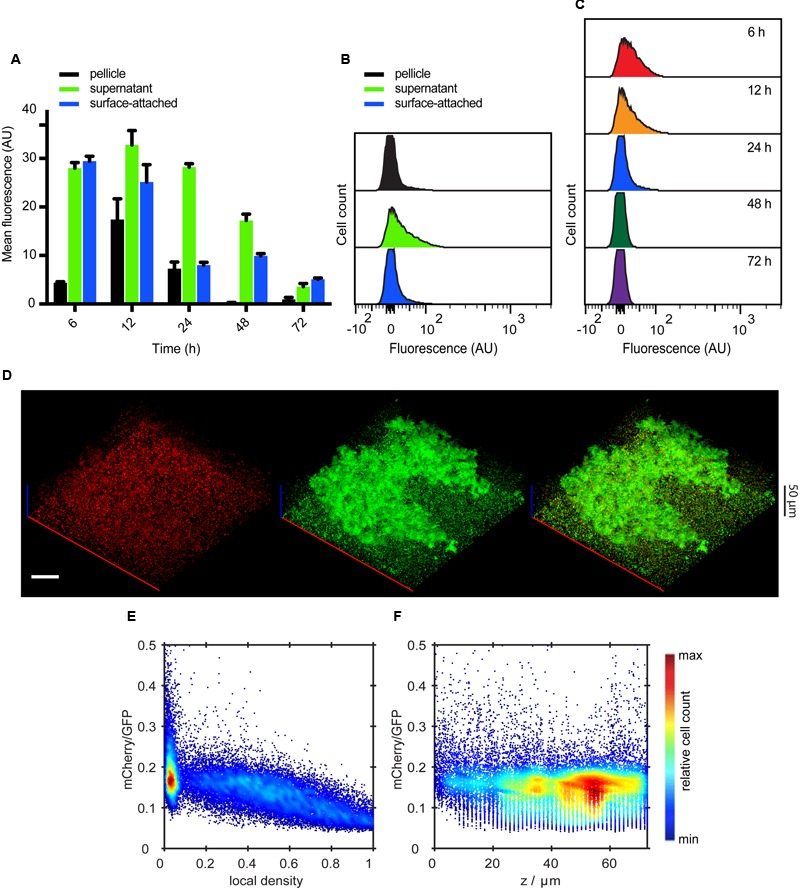
**Spatio-temporal expression of flagella in static submerged biofilms. (A)** Mean of P*fliC*-mCherry fluorescence intensity measured in indicated cell fractions by flow cytometry. Shown are mean values calculated from three independent experiments and standard errors. **(B)** Flow cytometry analysis of P*fliC*-mCherry fluorescence detected in individual biofilm fractions at 24 h. **(C)** Changes in the single-cell expression of flagella measured over time in surface-attached cells. **(D)** Confocal fluorescence microscopy images (maximum projections) showing expression of P*fliC*-mCherry (red) in biofilms at 24 h. All cells were labeled with constitutively expressed eGFP (green) (pVM42). Scale bar, 40 μm. **(E,F)** Expression profile of P*fliC*-mCherry as a function of local density **(E)** and along the height (*z*-axis) of the biofilm **(F)**. Values of mCherry fluorescence are normalized to fluorescence of eGFP which labels all cells. The color scale shows relative cell count from lowest (blue) to highest (red).

An opposite expression pattern was observed for P*fliC*-mCherry. After 12 h of incubation, the mean expression of flagella was decreasing over time in all three culture fractions, with highest expression being measured in the supernatant fraction (**Figures 3A,B**). Overall, this decrease in flagellin expression was monomodal and gradual over time (**Figures 3A,C**). Confocal microscopy showed that P*fliC*-mCherry was primarily expressed in single cells in the supernatant or at the surface, but not associated with structures (**Figure 3D**). Quantitative analysis of these images further confirmed that flagellin expression was mostly confined to the regions with low local densities (**Figure 3E**). The expression profile along the height of the submerged biofilm also revealed that the majority of cells expressing flagellin was above 40 μm from the bottom of the well (**Figure 3F**), i.e., in regions where mostly single cells are found (Supplementary Figure 1A).

**FIGURE 4:**
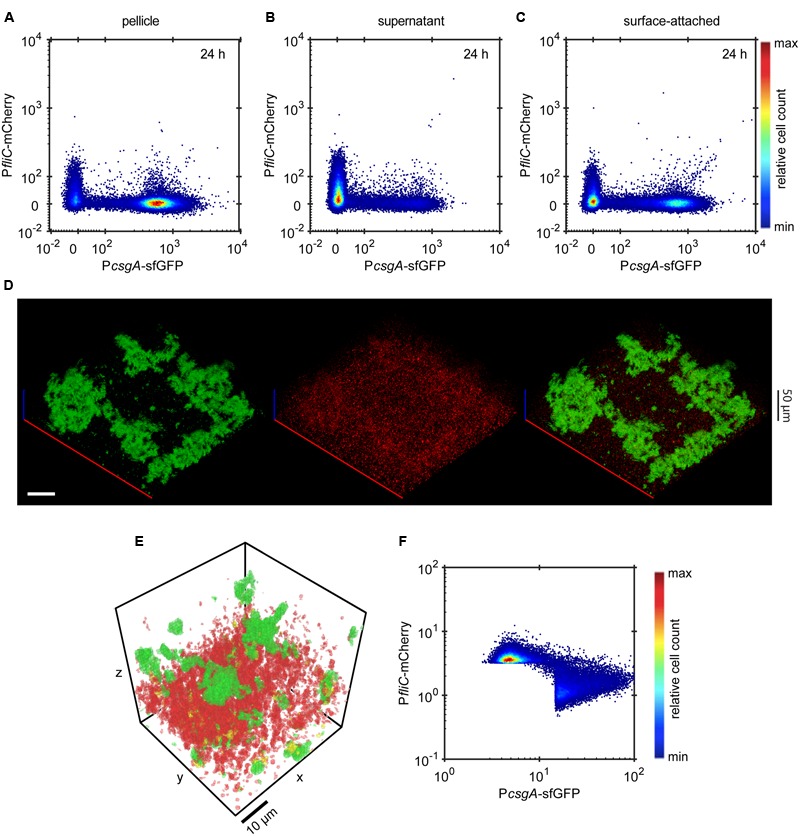
**Expression of curli fibers and flagella is mutually exclusive. (A-C)** Scatter plots showing expression of P*csgA*-sfGFP and P*fliC*-mCherry in **(A)** pellicle, **(B)** supernatant and **(C)** surface-attached cells at 24 h as measured by flow cytometry. The color scale shows relative cell count from lowest (blue) to highest (red). **(D)** Confocal fluorescence microscopy images (maximum projection) showing the expression of P*csgA*-sfGFP (green) and P*fliC*-mCherry (red) in biofilms at 24 h. Scale bar, 40 μm. **(E,F)** Quantification of relative expression of P*csgA*-sfGFP and P*fliC*-mCherry (see Material and Methods for details) in a region of the submerged biofilm shown in **(D)**, with a visual representation of the segmentation analysis **(E)** and relative expression in individual segments shown as a scatter plot **(F)**. In **(E)** green regions are P*csgA*-sfGFP-positive, red regions are P*fliC*-mCherry-positive and cells in yellow volumes express both fluorescence reporters. Note that sharp cutoffs in **(F)** correspond to the segmentation thresholds applied in image analysis (see Materials and Methods). The color scale shows relative cell count from lowest (blue) to highest (red).

To directly investigate the interplay between expression of curli and flagellar genes at a single-cell level, we created a double reporter strain that expresses both P*csgA*-sfGFP and P*fliC*-mCherry. Its analysis confirmed mutually exclusive expression of curli and flagella in all cell fractions and at all measured time points (**Figures 4A–C** and Supplementary Figure 2), with higher curli expression in the pellicle and in the surface-attached biofilm and with higher flagella expression in the supernatant fraction. Consistently, such mutually exclusive expression could also be seen (**Figure 4D**) for individual cells in intact submerged biofilms. This was confirmed by quantitative image analysis (**Figure 4E**) that clearly showed strictly exclusive expression of both reporters (**Figure 4F**).

### σ^S^ Activity Only Partly Correlates with Curli Expression

**FIGURE 5:**
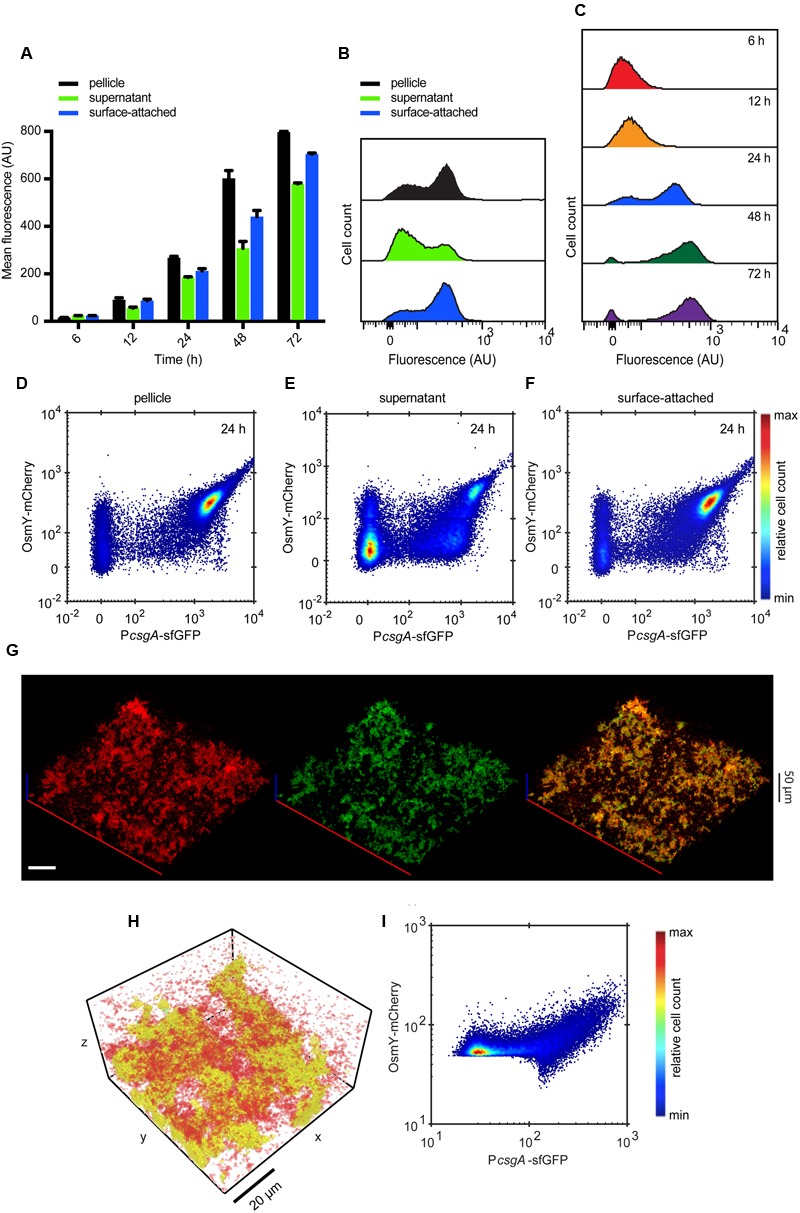
**Expression of curli fibers partly correlates with σ^S^ activity. (A)** Mean of OsmY-mCherry fluorescence intensity measured in the indicated culture fractions by flow cytometry. Shown are mean values calculated from three independent experiments and standard errors. **(B)** Flow cytometry analysis of OsmY-mCherry fluorescence detected in individual culture fractions at 24 h. **(C)** Changes in the single-cell expression of σ^S^ reporter measured over time in surface-attached cells. **(D-F)** Scatter plots showing expression of P*csgA*-sfGFP and OsmY-mCherry in **(D)** pellicle, **(E)** supernatant and **(F)** surface-attached cells at 24 h. The color scale shows relative cell count from lowest (blue) to highest (red). **(G)** Confocal fluorescence microscopy images (maximum projection) showing the expression of P*csgA*-sfGFP (green) and OsmY-mCherry (red) in biofilms at 24 h. Scale bar, 40 μm. **(H,I)** Quantification of relative expression of P*csgA*-sfGFP and OsmY-mCherry in a region of the submerged biofilm shown in **(G)**, with a visual representation of the segmentation analysis **(H)** and relative expression in individual segments shown as a scatter plot **(I)**. In **(H)** green regions are P*csgA*-sfGFP-positive, red regions are OsmY-mCherry-positive and cells in yellow volumes express both fluorescence markers. Note that sharp cutoffs in **(I)** correspond to the segmentation thresholds applied in image analysis (see Materials and Methods). The color scale shows relative cell count from lowest (blue) to highest (red).

To better understand the spatio-temporal pattern of curli expression, we studied the regulation of the stationary phase sigma factor, σ^S^ (Hengge-Aronis, 2002; Pesavento et al., 2008) during biofilm formation, using a translational fusion of mCherry to the σ^S^-regulated gene *osmY* as a reporter. The expression pattern of the σ^S^ reporter resembled that of curli, with a gradual increase over time and significantly higher expression in the pellicle and surface-attached biofilm fractions (*P* < 0.0001 according to an unpaired *t*-test) after 48 h of biofilm growth (**Figure 5A**). The expression of OsmY-mCherry was also bimodal in all biofilm fractions at 24 h of biofilm growth or later (**Figures 5B,C**). Furthermore, in all culture fractions we observed a strong linear correlation between *osmY* and curli expression in a subpopulation of cells (**Figures 5D–F**; Supplementary Figure 3). Nevertheless, we also detected a subpopulation of cells that express OsmY-mCherry but not P*csgA*-sfGFP. Most interestingly, a subpopulation of cells – particularly visible in the supernatant fraction at 24 h and in all fractions at 12 h – transiently expressed curli while having low σ^S^ activity, which indicates that curli expression switches to the ON state before full activation of σ^S^. All of these demonstrate that σ^S^ activity is only partly correlated with curli expression. Similarly, in biofilm images we observed curli expression specifically in biofilm structures and aggregates, whereas OsmY-mCherry was detected both in structures and in single cells (**Figures 5G,H**, Supplementary Figure 4). Quantitative image analysis (**Figure 5H**) confirmed partial correlation between curli expression and σ^S^ activity, with a subpopulation of cells showing high σ^S^ activity but low curli expression (**Figure 5I**). In summary, this analysis indicates that at least three distinct populations arise in submerged biofilms after 24 h of incubation: cells producing flagella, stationary cells producing curli and stationary cells not producing curli.

### Cells Within Biofilm Structures Show Reduced Growth

**FIGURE 6:**
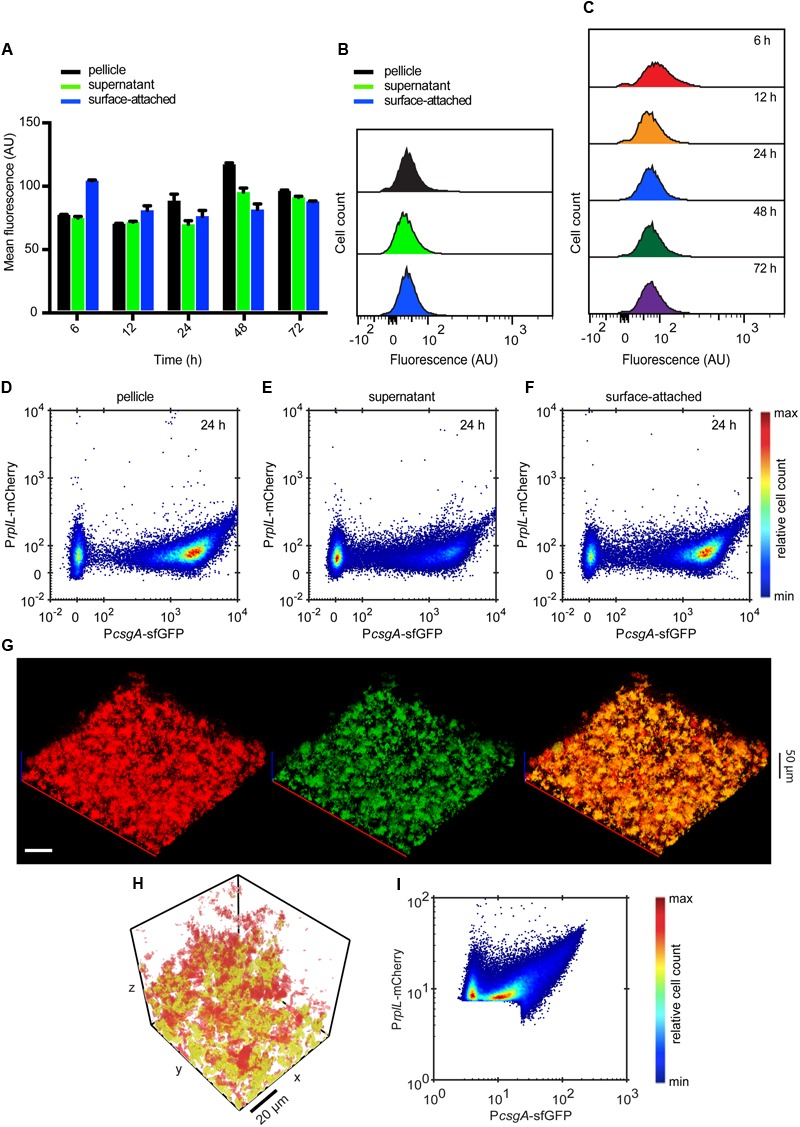
**Curli expression is independent of σ^D^ activity. (A)** Mean of P*rplL*-mCherry fluorescence intensity measured in indicated cell fractions by flow cytometry. **(B)** Flow cytometry analysis of P*rplL*-mCherry fluorescence detected in individual biofilm fractions at 24 h. **(C)** Changes in the single-cell expression of σ^D^ reporter measured over time in surface-attached cells. **(D-F)** Scatter plots showing expression of P*csgA*-sfGFP and P*rplL*-mCherry in **(D)** pellicle, **(E)** supernatant and **(F)** surface-attached cells at 24 h. The color scale shows relative cell count from lowest (blue) to highest (red). **(G)** Confocal fluorescence microscopy images (maximum projection) showing the expression of P*csgA*-sfGFP (green) and P*rplL*-mCherry (red) in biofilms at 24 h. Scale bar, 40 μm. **(H,I)** Quantification of relative expression of P*csgA*-sfGFP and P*rplL*-mCherry in a region of the submerged biofilm shown in **(G)**, with a visual representation of the segmentation analysis **(H)** and relative expression in individual segments shown as a scatter plot **(I)**. In **(H)** green regions are P*csgA*-sfGFP-positive, red regions are P*rplL*-mCherry-positive and cells in yellow volumes express both fluorescence markers. Note that sharp cutoffs in **(I)** correspond to the segmentation thresholds applied in image analysis (see Materials and Methods). The color scale shows relative cell count from lowest (blue) to highest (red).

Finally, we investigated the overall activity of the housekeeping sigma factor σ^D^ as well as the growth rate within *E. coli* biofilms in our open static culture system. A σ^D^-dependent transcriptional reporter P*rplL*-mCherry (*rplL* encodes the 50S ribosomal subunit L12) showed steady monomodal expression, with no pronounced change over time or difference between the biofilm fractions (**Figures 6A–C**). This confirms that housekeeping genes are similarly expressed in all cells across the population. Consistent with this observation, we found little correlation between the expression of P*rplL*-mCherry and P*csgA*-sfGFP (**Figures 6D–F**, Supplementary Figure 5). The same general pattern was observed within undisrupted submerged biofilms, with all cells expressing P*rplL*-mCherry irrespective of their association with the biofilm structures (**Figures 6G–I**). Thus there appears to be no significant correlation between curli expression and biosynthetic state of the cell.

**FIGURE 7:**
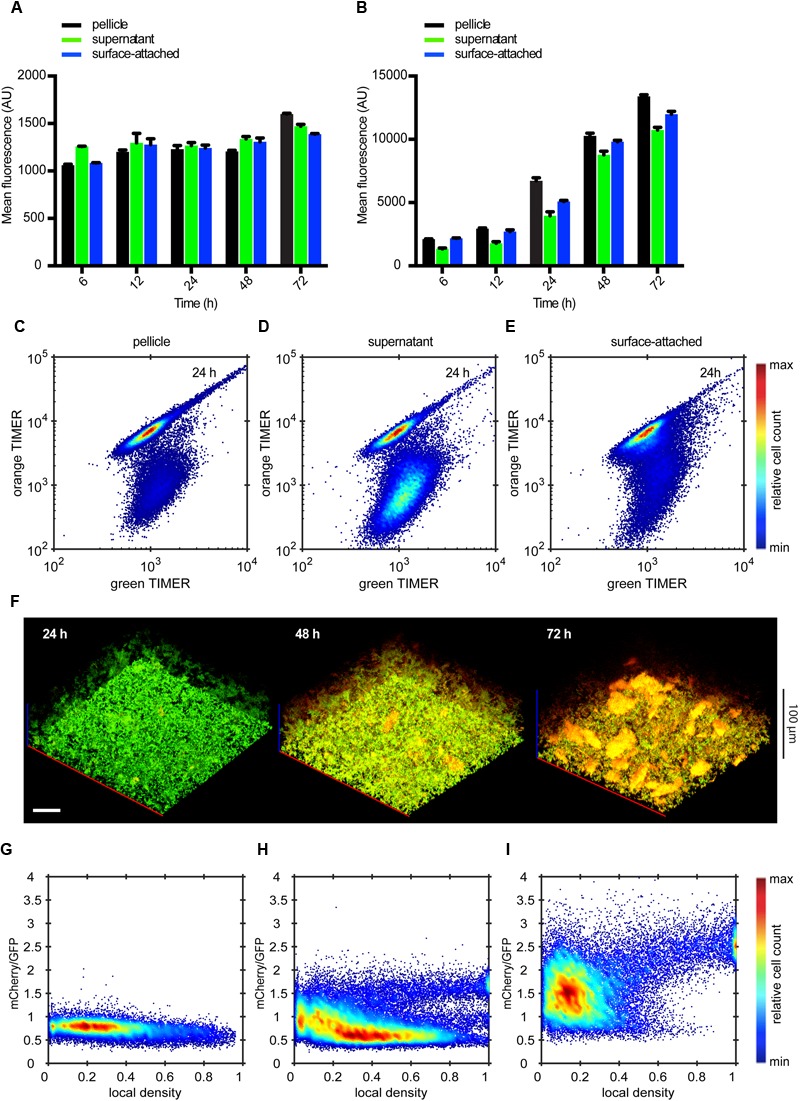
**Molecular timer analysis shows temporal accumulation of slow-growing cells in submerged static biofilms. (A-B)** Mean fluorescent intensity of fast-maturating green TIMER molecules **(A)** and slow-maturating orange TIMER molecules (B) in indicated cell fractions by flow cytometry. **(C-E)** Scatter plots showing expression of green TIMER molecules and orange TIMER molecules in **(D)** pellicle, **(E)** supernatant, and **(F)** surface-attached cells at 24 h. The color scale shows relative cell count from lowest (blue) to highest (red). **(F)** Confocal fluorescence microscopy images (maximum projection) showing fast-growing cells (green) and slow-growing cells (red) at 24, 48, and 72 h of biofilm growth (from right to left). Scale bar, 40 μm. **(G-I)** Expression profile of orange TIMER as a function of local density in submerged biofilms shown in **(F)** at 24 h **(G)**, 48 h **(H)**, and 72 h **(I)** of growth. Values of orange TIMER fluorescence are normalized to the internal control of green TIMER. The color scale shows relative cell count from lowest (blue) to highest (red).

A different pattern was observed for the single-cell growth rates. To monitor cell growth status in different biofilm fractions over time, we used a fluorescent growth rate reporter pOB44 (Claudi et al., 2014), which is based on a DsRed S197T variant called TIMER that spontaneously changes fluorescence from green to green/orange (Terskikh et al., 2000). Because green TIMER molecules have shorter maturation time than orange TIMER molecules, fast-growing cells that dilute both molecules with each cell division emerge green. In contrast, slowly growing cells accumulate both rapidly and slowly maturing TIMER molecules and appear green/orange, so that green/orange fluorescent ratios provide a readout for the growth rate. During biofilm development, we observed a stable green fluorescence and a gradual increase in red fluorescence in all fractions, with slightly higher values in the pellicle and in surface-attached cells (**Figures 7A,B**). A distinct subpopulation of slowly dividing cells apparently arises in the pellicle and surface-attached fractions already after 6 h of biofilm growth (Supplementary Figure 6) and later also in the supernatant fraction, gradually increasing with time (**Figures 7C–E**, Supplementary Figure 6). In biofilm structures, slowly proliferating cells were present almost exclusively in larger aggregates already at 24 h of growth and much more prominently at 72 h of growth (**Figure 7F**, Supplementary Figure 7). Importantly, rapidly dividing green cells were observed beneath and between aggregates of slowly dividing cells even at later time points. These observations were confirmed by quantification of the orange/green TIMER ratio, which showed both an overall increase throughout the growth of submerged biofilm and a particularly high level in a defined subpopulation of cells in the regions with highest local density (**Figures 7G–I**).

### Curli and Flagella Are Also Required for Biofilm Formation by the Cellulose-Producing *E. coli*

**FIGURE 8:**
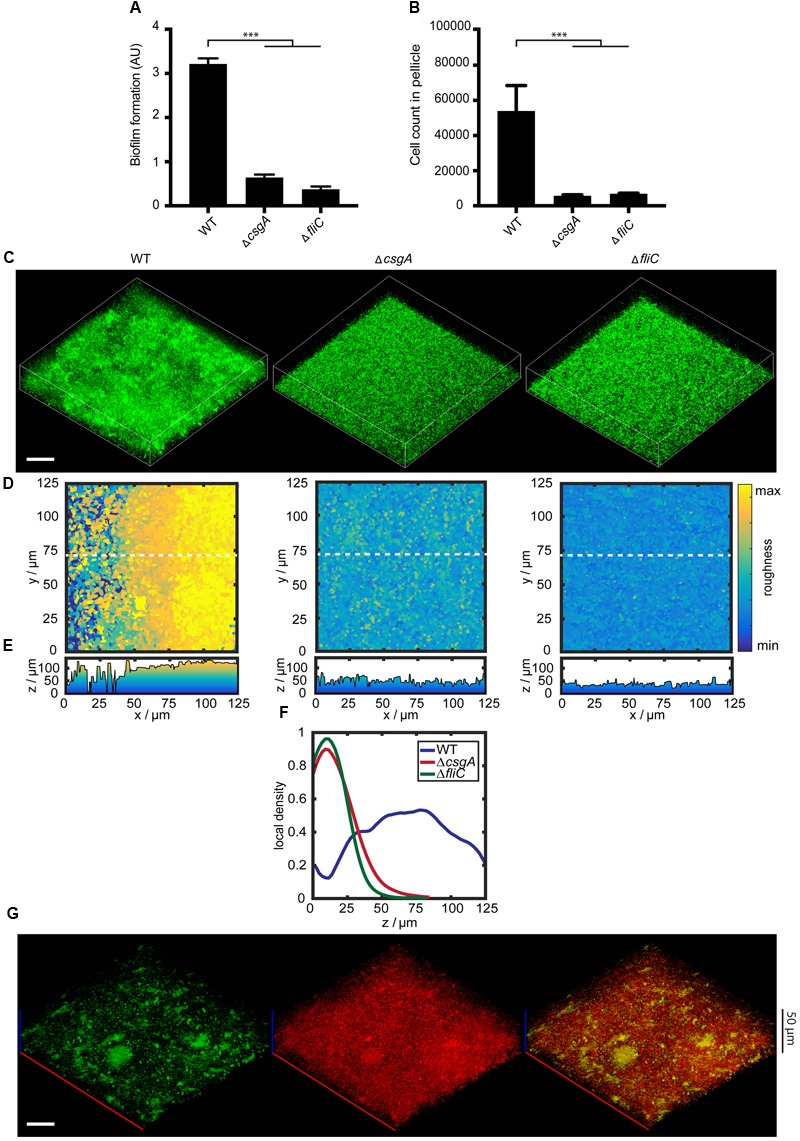
**Curli and flagella are required for biofilm structure development in de-domesticated strain AR3110. (A)** Biofilm formation by wild-type strain AR3110 and mutants lacking either curli (Δ*csgA*) or flagella (Δ*fliC*) that were grown under static conditions at 30°C for 24 h. Biofilm formation was quantified using CV staining, with CV values normalized to the optical density shown in arbitrary units (AU). ^∗∗∗^indicates *P* < 0.0001 according to unpaired *t*-test. Standard errors from three independent experiments are indicated. **(B)** Number of cells counted per 60 s in the pellicle fraction of submerged biofilms, measured using flow cytometry. **(C)** Confocal fluorescence microscopy images (maximum projection) of static biofilms grown in microtiter plates at 30°C for 24 h. All cells were labeled with eGFP as in **Figure 1D**. Scale bar, 40 μm. **(D)** Top views and corresponding **(E)** roughness profiles of submerged biofilms shown in **(C)**. The color scale shows roughness values from lowest (blue) to highest (yellow). **(F)** The profiles of mean local density throughout the biofilm height (*z*-axis) are shown in blue for W3110 (WT), in green for the mutant lacking flagella (Δ*fliC*) and in red for the mutant lacking curli (Δ*csgA*). **(G)** Confocal images (maximum projection) showing expression of P*csgA*-sfGFP (green) in static biofilms, grown in microtiter plates at 30°C for 24 h. For a reference, all cells were labeled with constitutively expressed mCherry (red) (pOB2). Scale bar, 40 μm.

Although *E. coli* K-12 normally does not produce cellulose, expression of cellulose genes in this strain can be reactivated (Serra et al., 2013a). We observed that the corresponding cellulose-producing strain AR3110 formed submerged biofilms and pellicles with the biomass comparable to that of W3110 (**Figure 8A**), showing that presence of cellulose as a matrix component does not generally enhance the biofilm formation. Similarly, deletion of either curli (Δ*csgA*) or flagella (Δ*fliC*) led to a decrease of biofilm and pellicle formation (**Figures 8A,B**) as well as of 3D structure (**Figures 8C–F**) in the AR3110 background, demonstrating that the presence of cellulose cannot compensate for the loss of curli matrix. The expression of P*csgA*-sfGFP within biofilm structures of the AR3110 strain was again confined to dense cell clumps and aggregates (**Figure 8G**). Nevertheless, biofilms formed by the AR3110 strain were more compact and had larger thickness than those of the W3110 strain (compare **Figure 8** with **Figure 1**), demonstrating that cellulose does contribute to the overall structure of the submerged static biofilms.

## Discussion

Biofilm formation is a highly regulated, complex, and dynamic process. Recent studies have demonstrated that distinct subpopulations may emerge during bacterial biofilm development, which differ with respect to their stress resistance, motility, production of extracellular matrix components, and growth status (Häussler, 2004; Hansen et al., 2007; Chai et al., 2008; Bernier et al., 2013; Guttenplan and Kearns, 2013; Serra and Hengge, 2014; van Gestel et al., 2015). However, current understanding of the spatio-temporal dynamics of gene expression and cell differentiation within biofilms is still very limited, even for model organisms such as *E. coli*, and our work provides one of the first insights into such regulation at the single-cell level.

Here we focused on submerged biofilms formed by *E. coli* strain W3110 under static conditions at 30°C, one of the first laboratory biofilm models (Pratt and Kolter, 1998). In agreement with previous studies of *E. coli* biofilms (Pratt and Kolter, 1998; Römling et al., 1998a; Hung et al., 2013; Serra et al., 2013b), our CV and confocal microscopy analysis revealed that curli and flagella are crucial for *E. coli* biofilm formation under these conditions, apparently playing a role both in the surface attachment and in subsequent biofilm structure development. Among other tested putative adhesins and matrix components, only the deletion of fimbriae showed a major effect on biofilm formation, affecting surface attachment but not biofilm structure. Notably, we observed that curli and flagella were also required for pellicle formation at the liquid-air interface. Since the same components were shown to play a key structural role in macrocolony biofilms (Serra et al., 2013b), our results demonstrate similarity between all types of *E. coli* biofilms.

The observed requirement of curli and flagella for three-dimensional biofilm structure formation was also confirmed in the cellulose-producing strain AR3110 (Serra et al., 2013a). Moreover, the overall numbers of cells within submerged biofilms and pellicles formed by the AR3110 and W3110 strains was similar. Nevertheless, biofilms formed by the AR3110 strain were more compact, showing that whereas cellulose cannot substitute for curli as a major matrix component, it does contribute to the overall biofilm structure.

Although flagella and curli are apparently both necessary for biofilm formation, we observed that the expression levels of genes encoding these cellular structures were anti-correlated in time, with increasing curli and decreasing flagella expression over the course of biofilm development. While this is consistent with previously proposed regulation during the transition from motile towards sessile lifestyles (Pesavento et al., 2008), we showed that this mutually exclusive regulation of flagella and curli can be observed even at the single cell level, meaning that curli-producing cells shut down the production of flagella and vice versa. Consistent with this observation, curli- and flagella-producing cells were detected in different cell fractions and regions within the submerged biofilms. Within submerged biofilms, curli fibers are highly expressed in regions of high local density, corresponding to pellicles, cell aggregates and microcolonies, but not in individual cells. In contrast, flagella show the opposite expression pattern, expressed only in single cells between aggregates and in direct contact to the surface. Although in *E. coli* macrocolony biofilms curli- and flagella-producing cells were also observed in distinct zones of the biofilm, this segregation was proposed to be due to the global changes in gene expression in response to nutrient availability (Serra et al., 2013b). However, in our biofilm system, the observed cellular differentiation is unlikely to be driven by nutrient gradients, given the porous structure of *E. coli* submerged biofilms. Importantly, we detected two distinct subpopulations of curli-ON and curli-OFF cells throughout the time course of biofilm development, comparable to observations made for CsgD in a previous study (Grantcharova et al., 2010).

Although the observed expression of the σ^S^ reporter OsmY-mCherry was also bimodal, our results showed that it only correlates with curli expression in a subpopulation of cells, as a large fraction of cells shows active σ^S^ transcription but no curli expression. In addition, the bimodality in the activity of σ^S^ was observed only from 24 h onward, i.e., after appearance of the curli-ON fraction. Even more intriguingly, we also observed a minor subpopulation of cells with high curli expression but low σ^S^ activity. These results suggest that bimodality in curli expression arises due to the regulation downstream from σ^S^, most probably at the level of CsgD (Grantcharova et al., 2010; Yousef et al., 2015), and that the curli-ON switch can be achieved already prior to the full activation of σ^S^. Notably, *csgD* expression is either directly or indirectly regulated by a number of cellular factors, including different transcription factors, levels of second messenger cyclic diguanosine monophosphate (cyclic di-GMP) and small regulatory RNAs (sRNAs) that mediate cellular responses to diverse environmental changes (Gerstel and Römling, 2003; Jubelin et al., 2005; Pesavento and Hengge, 2009; Ishihama, 2010; Ogasawara et al., 2010a; Liu et al., 2014; Michaux et al., 2014; Mika and Hengge, 2014). For example, the sRNAs ArcZ, RprA, and DsrA have been shown to modulate activity of σ^S^ in response to oxygen availability, membrane stress, temperature and pH, respectively (Monteiro et al., 2012; Mika and Hengge, 2014). The possible roles of cyclic di-GMP and sRNA-dependent regulation in the observed diversification of gene expression during formation of submerged *E. coli* biofilms require further investigation.

Finally, we could also demonstrate clear differences in the growth rate of cells within different subpopulations during formation of submerged static biofilms. We found that at later stages of biofilm development, cells that are associated in aggregates reduce their growth. In contrast, no apparent differences could be observed in the expression of housekeeping genes, and thus presumably in the cell metabolic activity, across the biofilm. Interestingly, in mature biofilms, slowly dividing cells in aggregates coexist with faster dividing single cells, despite identical nutritional environment and likely similar levels of σ^S^ activity. This suggests that cells associated in structures might actively slow down their proliferation, following a specific developmental program. Since we could not observe any apparent gradient of the growth rate within cellular aggregates, it seems unlikely that this reduction of growth is caused by the nutritional or other gradients.

Altogether, our results demonstrate the existence of at least three specialized cell subpopulations in static submerged biofilms of *E. coli*, which are distinctly different in their gene expression profiles and presumably in their physiological state. Central for the 3D structure formation are stationary cells with high levels of σ^S^ activity and curli production, which are almost exclusively associated in aggregates within the biofilm or the pellicle and show reduced growth in mature biofilms. This biofilm-forming population coexists with a subpopulation of stationary cells that also have high levels of σ^S^ activity but do not produce curli and are not associated in aggregates, but apparently continue growing. Finally, even in mature biofilms we observe a subpopulation of apparently post-exponential single cells that produce flagella and reside outside of the aggregates, even though this subpopulation decreases with time. Although the physiological importance of this heterogeneity and its regulation by the environmental and growth conditions needs further investigation, the observed diversification appears to be spontaneous rather than being driven by nutrient or other gradients as proposed in previous studies (Boles et al., 2004; Klausen et al., 2006; Grantcharova et al., 2010; Arnoldini et al., 2012), and might thus represent a form of bet hedging behavior (Veening et al., 2008).

## Author Contributions

OB and VS designed the experiments. OB and VMS performed the experiments. RH, OB, and KD performed the quantitative analysis. OB and VS analyzed the data and wrote the manuscript.

## Conflict of Interest Statement

The authors declare that the research was conducted in the absence of any commercial or financial relationships that could be construed as a potential conflict of interest.
