# Supplementary material for: Diversification of Gene Expression during Formation of Static Submerged Biofilms by Escherichia coli
Source: Front Microbiol. 2016 Oct 5;7:1568. doi: 10.3389/fmicb.2016.01568 (PMC5050211; doi:10.3389/fmicb.2016.01568)
Supplement: Supplementary file 1 [file Table_1.pdf]

Supplementary Table 1. ***E. coli*** **strains and plasmids used in this study.**

Strains	Relevant genotype or phenotype[a]	Reference
W3110	W3110 derivative with functional RpoS (*rpoS396(Am)*)	(Serra et al., 2013a)
AR3110	W3110 derivative with functional cellulose synthase *bcsQ*	(Serra et al., 2013b)
VM388	W3110 Δ*fliC* Km^s	This work
VM392	W3110 Δ*csgA*::Km^r	This work
VM335	AR3110 Δ*fliC*::Km^r	This work
VM661	AR3110 Δ*csgA* Km^s	This work
VM391	W3110 Δ*pgaC* Km^s	This work
VM482	W3110 Δ*wcaF*::Km^r	This work
VS824	W3110 Δ*flu* Km^s	This work
OB301	W3110 Δ*fimA*::Km^r	This work
OB148	W3110 Δ*sfmH*::Km^r	This work
OB153	W3110 Δ*ybgP*::Km^r	This work
OB154	W3110 Δ*yraH*::Km^r	This work
OB156	W3110 Δ*yehD*::Km^r	This work

OB166	W3110 ΔypjA::Km^r	This work
OB135	W3110 *fliC*::*fliC-RBS-mCherry codon optimized* Km^s	This work
OB136	W3110 c*sgA*::*csgA-RBS-sfGFP codon optimized* Km^s	This work
OB53	W3110 *csgA*::*csgA-RBS-sfGFP* Km^s*, fliC*::*fliC-RBS-mCherry opt* Km^r	This work
OB54	W3110 *csgA*::*csgA-RBS-sfGFP* Km^s*, osmY-mCherry* Km^r	This work
OB55	W3110 *csgA*::*csgA-RBS-sfGFP* Km^s*, rplL*::*rplL-RBS-mCherry opt* Km^r	This work
OB191	AR3110 c*sgA*::*csgA-RBS-sfGFP codon optimized* Km^r	This work

Plasmids	Relevant genotype or phenotype[a]	Reference
pTrc99a	Amp^r; Expression vector; pBR ori; pTrc promoter; IPTG-inducible	(Amann et al., 1988)
pVM42	Amp^r; *eGFP* in pTrc99a, IPTG-inducible	This work
pOB2	Amp^r; *mCherry* in pTrc99a, IPTG-inducible	This work
pOB44	Amp^r; TIMER^{bac} in pBR322	(Claudi et al., 2014)
pCP20	Amp^r; provides *flp* recombinase required for Km^r removal	(Cherepanov and Wackernagel, 1995)

[a] Amp^r and Km^r indicate ampicillin and kanamycin resistance, respectively; Km^s indicate kanamycin sensitivity.

Supplementary references:

Amann, E., Ochs, B., and Abel, K. J. (1988). Tightly regulated tac promoter vectors useful for the expression of unfused and fused proteins in *Escherichia coli*. *Gene 69*, 301–315.

Cherepanov, P. P., and Wackernagel, W. (1995). Gene disruption in *Escherichia coli*: TcR and KmR cassettes with the option of Flp-catalyzed excision of the antibiotic-resistance determinant. *Gene 158*, 9–14.

Claudi, B., Spröte, P., Chirkova, A., Personnic, N., Zankl, J., Schürmann, N., Schmidt, A., and Bumann, D. (2014). Phenotypic Variation of *Salmonella* in Host Tissues Delays Eradication by Antimicrobial Chemotherapy. *Cell 158*, 722–733.

Serra, D. O., Richter, A. M., Klauck, G., Mika, F., and Hengge, R. (2013a). Microanatomy at cellular resolution and spatial order of physiological differentiation in a bacterial biofilm. *MBio 4*. e103-e113. doi: 10.1128/mBio.00103-13

Serra, D. O., Richter, A. M., and Hengge, R. (2013b). Cellulose as an architectural element in spatially structured *Escherichia coli* biofilms. *J. Bacteriol. 195*, 5540–5554.
