# Supplementary material for: Diversification of Gene Expression during Formation of Static Submerged Biofilms by Escherichia coli
Source: Front Microbiol. 2016 Oct 5;7:1568. doi: 10.3389/fmicb.2016.01568 (PMC5050211; doi:10.3389/fmicb.2016.01568)
Supplement: Supplementary file 2 [file Image_1.PDF]

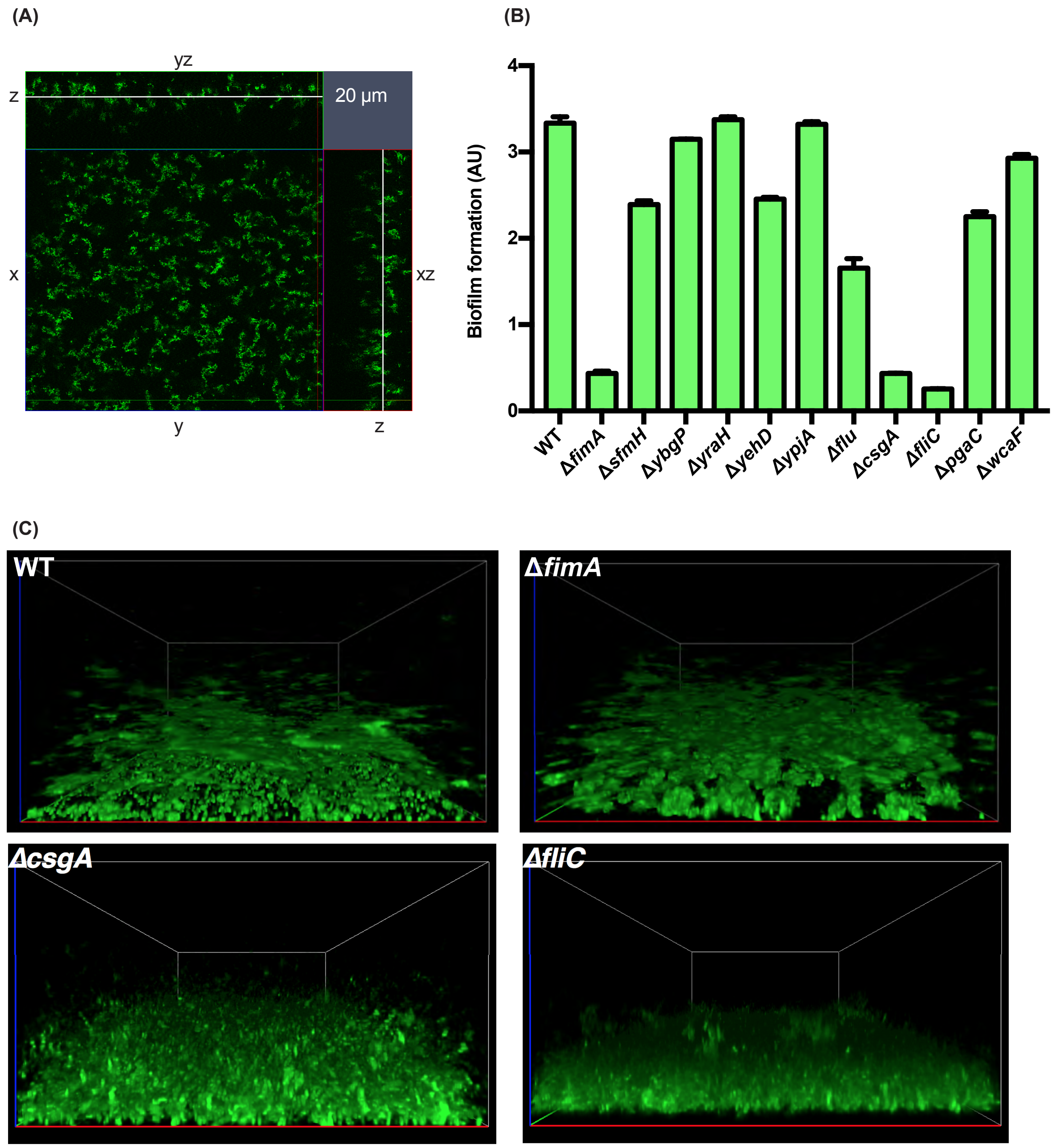

Supplementary Figure 1. Role of putative adhesins and matrix components in *E. coli* biofilm formation. (A) Confocal fluorescence microscopy image of the submerged biofilm formed by wild-type *E. coli* cells grown in microtiter dishes at 30°C for 24 h. Shown is an ortho-view at 20 µm from the bottom of the well. All cells were labeled with constitutively expressed eGFP (green) (pVM42). Scale bar, 20 µm. **(B)** Biofilm formation by the wild-type strain W3110 and mutants lacking fimbriae (Δ*fimA*), putative chaperone-usher adhesins (Δ*sfmH*, Δ*ybgP*, Δ*yraH*, Δ*yehD*), Antigen 43 (Δ*flu*), a protein with similarity to Antigen 43 (Δ*ypjA*), curli fibers (Δ*csgA*), flagella (Δ*fliC*), PGA (Δ*pgaC*) or colanic acid (Δ*wcaF*) that were grown under static conditions at 30°C for 24 h. Biofilm formation was quantified using crystal violet (CV) staining, with CV values normalized to the optical density shown in arbitrary units (AU). Standard errors from two independent experiments are indicated. **(C)** Confocal laser scanning microscopy images of static biofilms of indicated mutants grown in microtiter plates at 30°C for 48 h. Dimensions of bounding box (x:y:z): 142:142:80 µm.
