# Supplementary material for: Diversification of Gene Expression during Formation of Static Submerged Biofilms by Escherichia coli
Source: Front Microbiol. 2016 Oct 5;7:1568. doi: 10.3389/fmicb.2016.01568 (PMC5050211; doi:10.3389/fmicb.2016.01568)
Supplement: Supplementary file 4 [file Image_3.pdf]

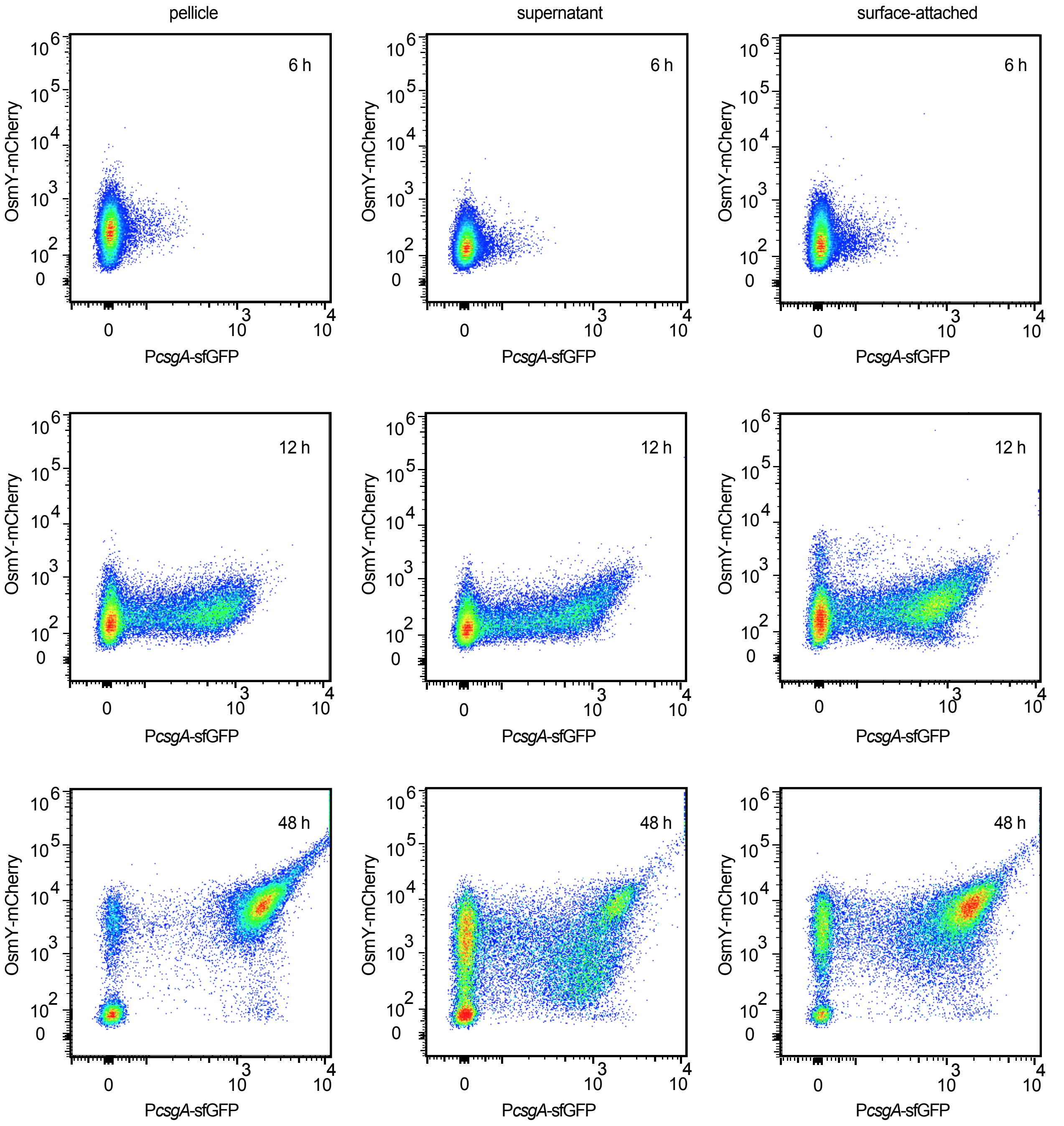

Supplementary Figure 3. Expression of curli fibers only partially correlates with σ^S activity throughout the biofilm growth. Scatter plots showing expression of P*csgA*-sfGFP and OsmY-mCherry in pellicle, supernatant and surface-attached cells at selected time points by flow cytometry. Expression levels of P*csgA*-sfGFP and σS-regulated OsmY-mCherry are plotted on the *x*-axis and *y*-axis, respectively. The color scale is as in Figure 4.
