# Supplementary material for: Diversification of Gene Expression during Formation of Static Submerged Biofilms by Escherichia coli
Source: Front Microbiol. 2016 Oct 5;7:1568. doi: 10.3389/fmicb.2016.01568 (PMC5050211; doi:10.3389/fmicb.2016.01568)
Supplement: Supplementary file 5 [file Image_4.pdf]

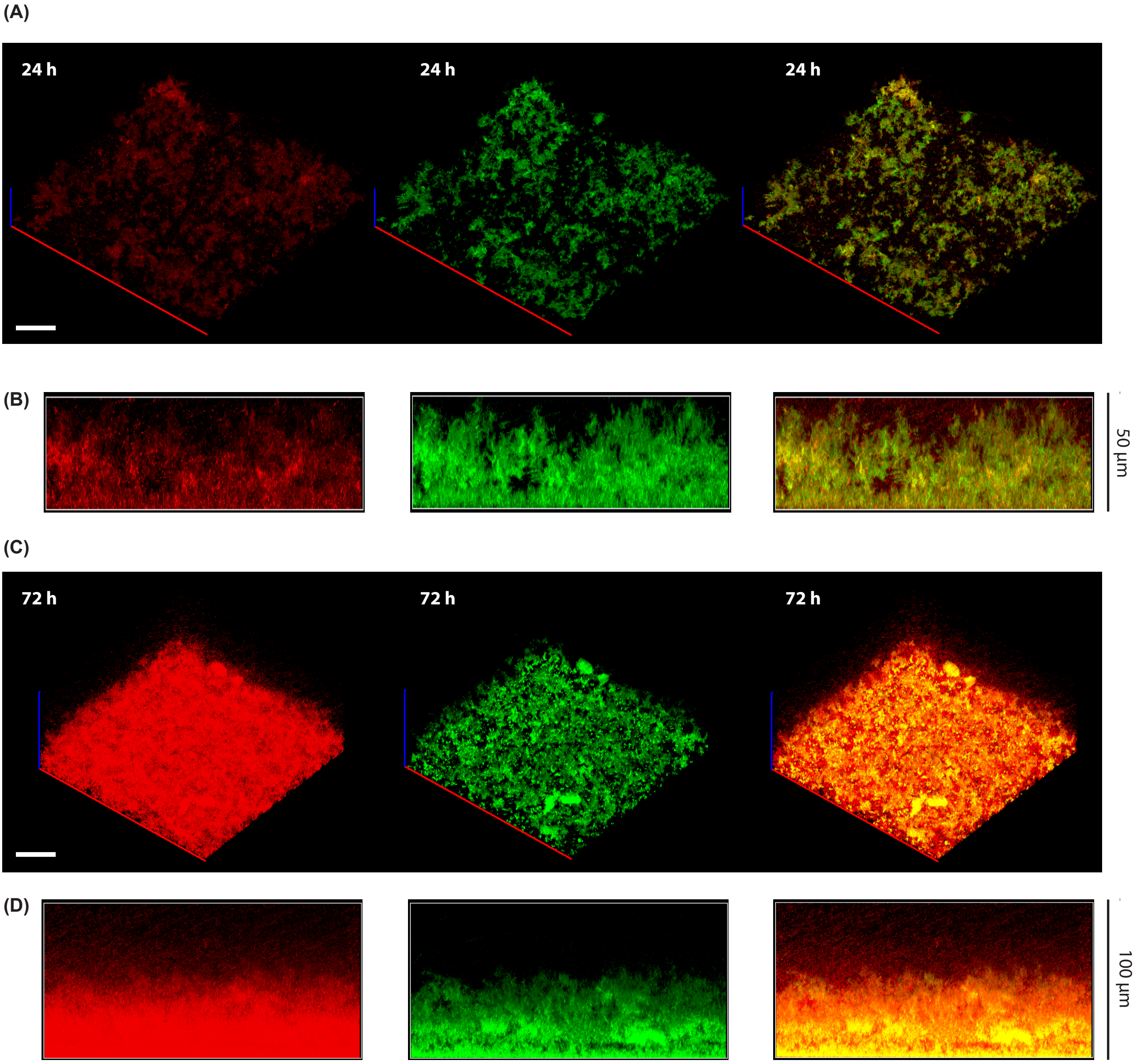

Supplementary Figure 4. Restriction of curli expression to biofilm microcolonies cannot be explained by σ^S activity. A strong increase in σ^S activity can be seen between 24 h **(A-B)** and 72 h **(C-D)** of incubation in all biofilm cells, independent on the localization within the biofilm. Scale bars: 40 µm.
