# Supplementary material for: Diversification of Gene Expression during Formation of Static Submerged Biofilms by Escherichia coli
Source: Front Microbiol. 2016 Oct 5;7:1568. doi: 10.3389/fmicb.2016.01568 (PMC5050211; doi:10.3389/fmicb.2016.01568)
Supplement: Supplementary file 6 [file Image_5.pdf]

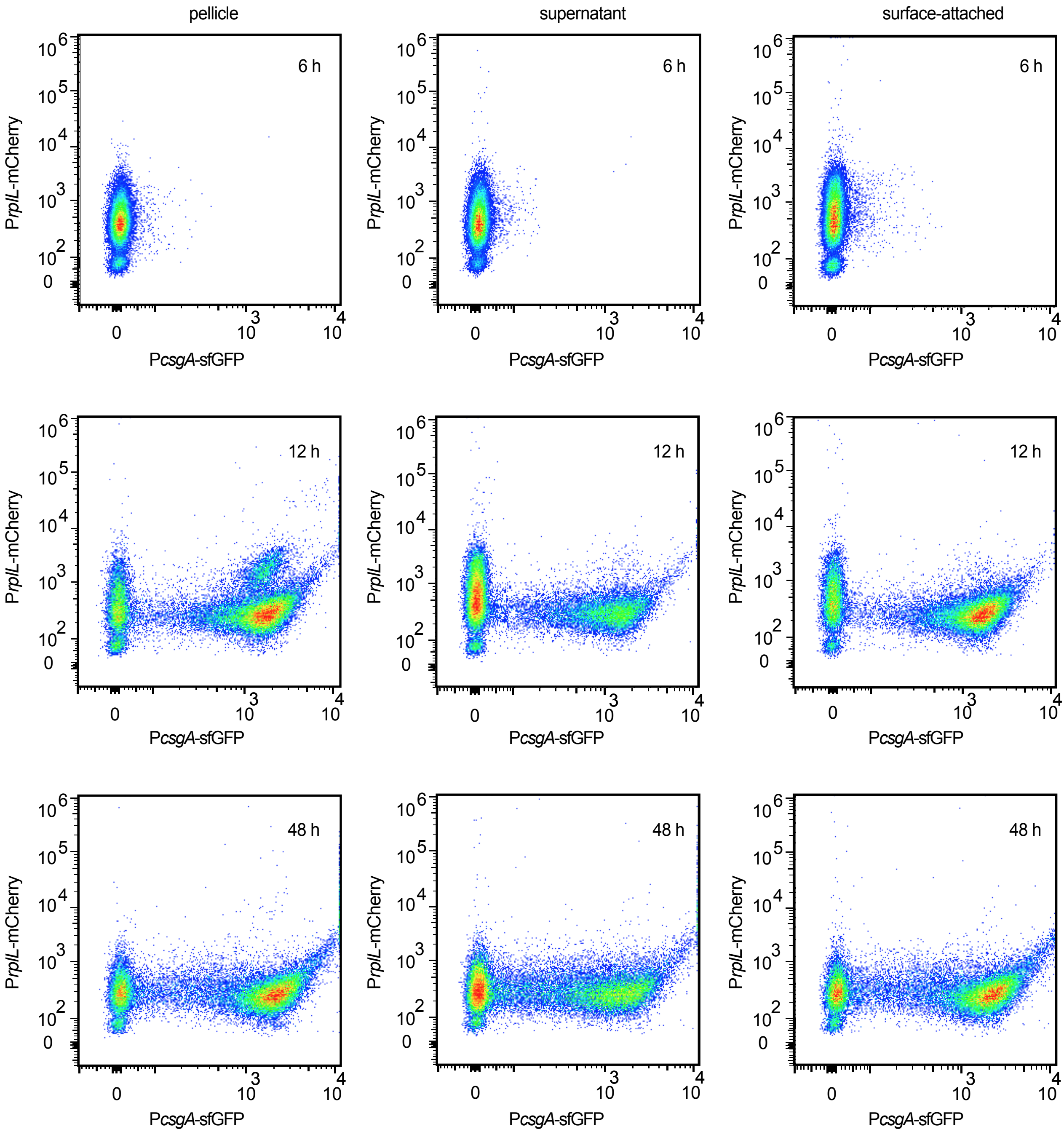


**Supplementary Figure 5. All cells in biofilm show similar σ$^D$ activity irrespective of curli expression.** Scatter plots showing expression of P*csgA*-sfGFP and P*rplL*-mCherry in pellicle, supernatant and surface-attached cells at selected time points by flow cytometry. Expression levels of P*csgA*-sfGFP and σ$^D$-regulated P*rplL*-mCherry are plotted on the *x*-axis and *y*-axis, respectively. The color scale is as in Figure 4.
