# Supplementary material for: Diversification of Gene Expression during Formation of Static Submerged Biofilms by Escherichia coli
Source: Front Microbiol. 2016 Oct 5;7:1568. doi: 10.3389/fmicb.2016.01568 (PMC5050211; doi:10.3389/fmicb.2016.01568)
Supplement: Supplementary file 7 [file Image_6.PDF]

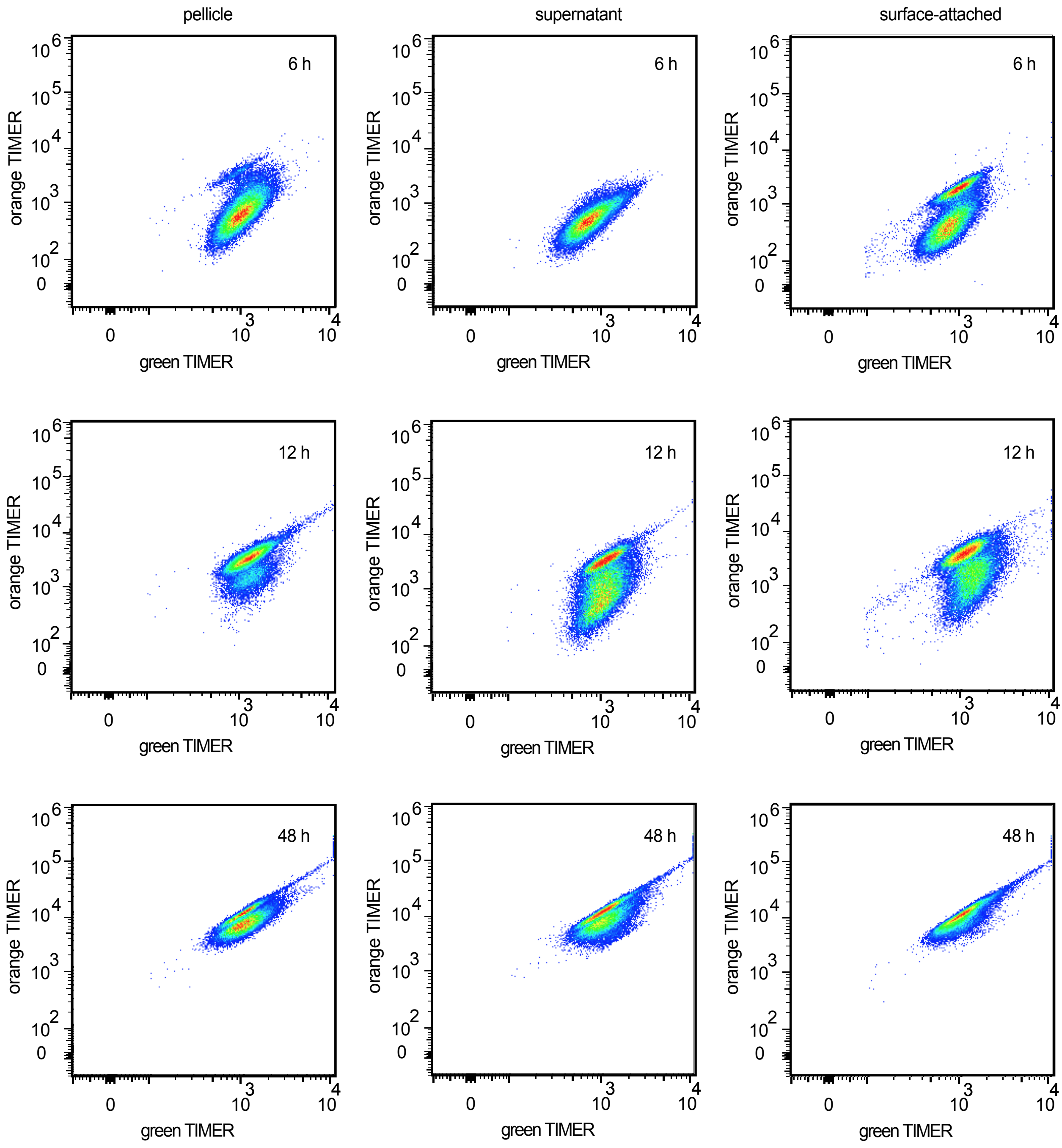


**Supplementary Figure 6. Slowly dividing cells accumulate during biofilm growth.** Scatter plots showing expression of green TIMER molecules and orange TIMER molecules in pellicle, supernatant and surface-attached cells at selected time points, measured by flow cytometry. Expression of green TIMER molecules (fast-dividing cells) and orange TIMER molecules (slow-diving cells) are plotted on the *x*-axis (green TIMER) and *y*-axis (orange TIMER), respectively. The color scale is as in Figure 4.
