# Supplementary material for: Diversification of Gene Expression during Formation of Static Submerged Biofilms by Escherichia coli
Source: Front Microbiol. 2016 Oct 5;7:1568. doi: 10.3389/fmicb.2016.01568 (PMC5050211; doi:10.3389/fmicb.2016.01568)
Supplement: Supplementary file 8 [file Image_7.PDF]

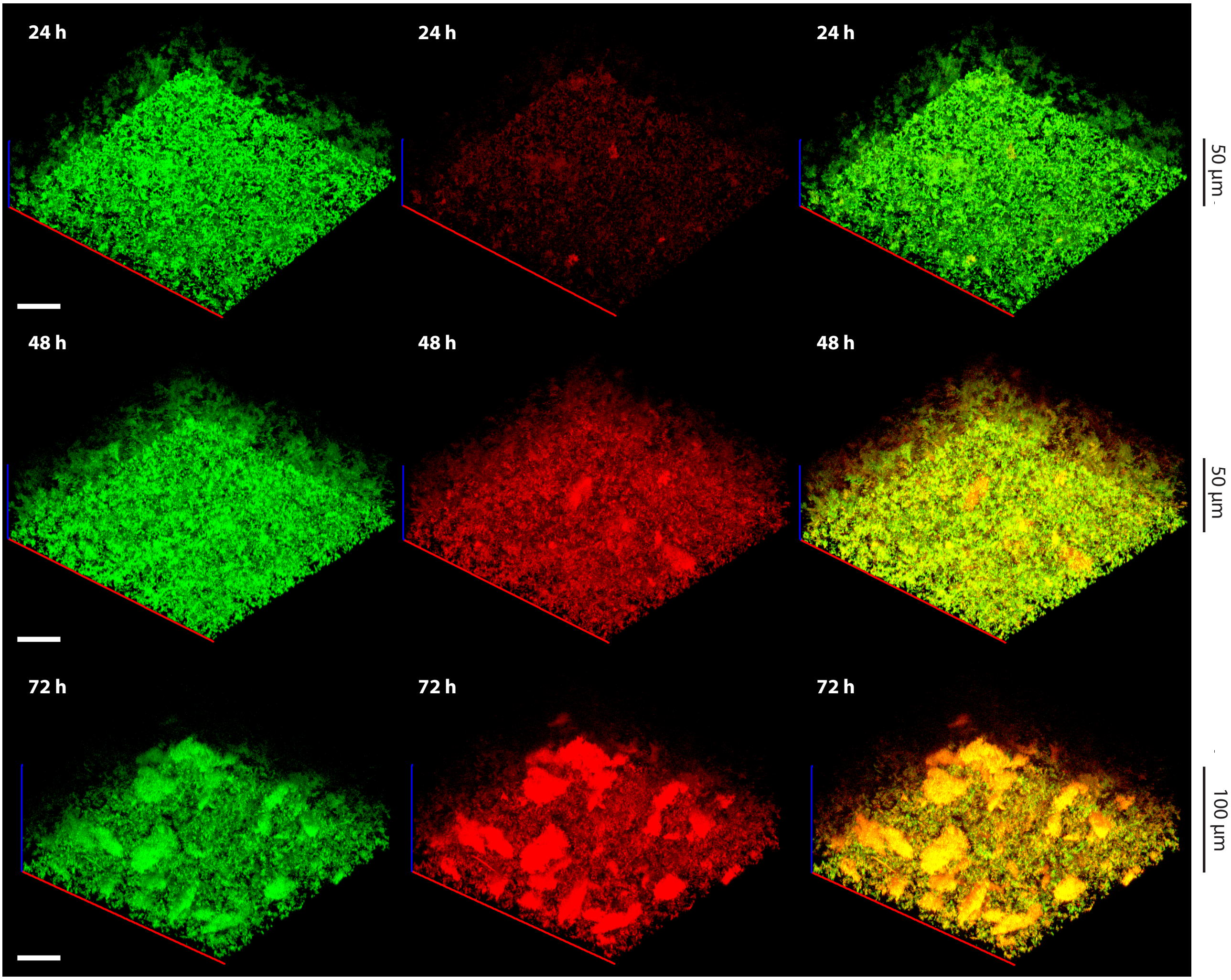

Supplementary Figure 7. Confocal fluorescence microscopy reveals accumulation of slowly dividing cells with time specifically in dense cellular structures. Orange TIMER molecules (red signal), which indicate slowly proliferating cells, accumulate between 24 and 72 h of biofilm growth. Scale bars, 40 µm.
