# Supplementary material for: Diversification of Gene Expression during Formation of Static Submerged Biofilms by Escherichia coli
Source: Front Microbiol. 2016 Oct 5;7:1568. doi: 10.3389/fmicb.2016.01568 (PMC5050211; doi:10.3389/fmicb.2016.01568)
Supplement: Supplementary file 9 [file Image_8.PDF]

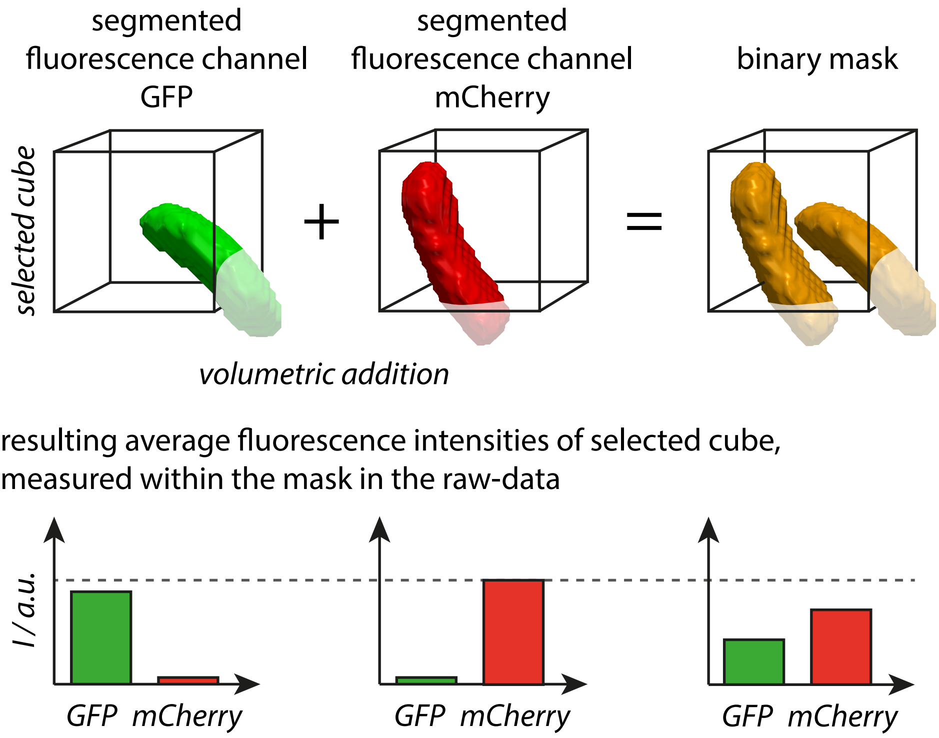

Supplementary Figure 8. Segmentation of cells without constitutive fluorescence marker. For a hypothetical cube bearing a GFP- and an mCherry-positive cell, image segmentation of the GFP channel yields the volumetric information of the green cell, whereas segmentation of the mCherry channel yields the volumetric information of the red cell. Prior to intensity measurements, the volumetric information from both channels are merged and the binary mask indicated in yellow is obtained. Now both, the average green and red fluorescence intensity are lower compared to the case where either only the red or green cell was present because the binary mask is including voxels with intensity below the segmentation threshold used for the particular fluorescence channel. Following this procedure, individual intensity values in scatter plots (Fig. 4F, Fig. 5I and Fig. 6I) can show intensities below the segmentation threshold.
